# Survey data on inter-firm linkages and innovation activities of Chinese manufacturing SMEs

**DOI:** 10.1016/j.dib.2019.104671

**Published:** 2019-10-16

**Authors:** Liang Mei, Tao Zhang, Jin Chen

**Affiliations:** a National School of Development, Peking University, Beijing, 100871, China; b Loughborough University London, Here East, Queen Elizabeth Olympic Park, London, E15 2GZ, UK; c Research Centre for Technological Innovation, Tsinghua University, Beijing, 100084, China; d School of Economics and Management, Tsinghua University, Beijing, 100084, China

**Keywords:** Inter-firm linkages, Innovation performance, Absorptive capacity, Survey

## Abstract

The data presented in this article is based on a questionnaire survey regarding the inter-firm linkages and innovation activities of Chinese manufacturing SMEs. A valid sample of 420 SMEs was collected, covering six manufacturing industries. The data involves the inter-firm linkages, innovation performance, R&D intensity, IT adoption, and other demographic indicators of the sampling firms. In this data article, we also briefly present the subject area, background of data source, survey process, scale of data, value of data, and main methods used to analyze the data.

**Table:** Specifications Table

Subject area	Business, Management and Accounting
More specific subject area	Management of Technology and Innovation
Type of data	Table
How data was acquired	Survey
Data format	Raw and Filtered
Experimental factors	1. A full-sample data set was provided containing 420 valid sampling firms for analysis, involving items such as firm ownership, firm scale, firms' export orientation, industrial classification, product innovation performance, and evaluation of external linkages. 2. A sub-sample data set covering 319 medium-sized sampling firms was provided for extra study.
Experimental features	1. Firm ownership was calculated by the item (Private), firm scale was calculated by the item (Person) with the item (lnperson) be transferred by natural logarithm algorithm, firms' export orientation was measured by the item (export). 2. IP was calculated by set (p1-p4), representing the innovation performance of firms. 3. LPO presented in the full model was calculated by set (a1-a4), representing the linkages with competitors, suppliers, lead users and customers, and complementors; LSI was calculated by set (a5-a8), representing the linkages with universities, research institutes, governance agencies, and finance and law service agencies. 4. AC in one model was calculated by standardized value of R&D expenditures (represented by ZRD), and in the other mode directly calculated by the item (IT), indicating the firm engaging information technology activities or not. 5. The item (industry) represented the industry background of samples. 6. Structural Equation Model was adopted for data analysis via statistical software AMOs.
Data source location	Zhejiang Province, China
Data accessibility	With this article
Related research article	Mei, L., Zhang, T. and Chen, J. 2019. Exploring the effects of inter-firm linkages on SMEs' open innovation from an ecosystem perspective: An empirical study of Chinese manufacturing SMEs. Technological Forecasting and Social Change, 144, pp.118–128. DOI: https://doi.org/10.1016/j.techfore.2019.04.010

**Table:** 

**Value of the Data** - The survey could serve as a database contributing knowledge on China's manufacturing SMEs. As SMEs' data is difficult to access in general channels like statistical yearbooks of China, the survey provided a template for Chinese SMEs data collection, contributing to the research on SMEs' orientation for external innovation linkages, internal innovation management, and performance. - The survey and relevant data analysis process could serve as a reference facilitating comparative studies on SMEs' innovations among other geographical countries' contexts. Prior literature has provided enriched evidences on innovation activities from enterprises in Europe via survey, such as the Community Innovation Survey (CIS) of EU. However, data related to Chinese enterprises is scarce, let alone the SMEs. Therefore, the date could be regarded as such references to complement the Chinese evidences for global innovation research. - The two measures on absorptive capacity, i.e. R&D intensity measure and IT adoption measure, are useful for future absorptive capacity research, and the measure of inter-firm linkages can be used in future open innovation research.

## 1 Data

The data described in the paper provides information about manufacturing firms' innovation activities in a particular region - Zhejiang Province - of China, a region where SMEs and relevant private firms are the economic pillars. Data was collected by a sample survey, with the questionnaires and relevant items constructed according to prior innovation literature. Statistical methods based upon large scale sample data were adopted to analyze both full-sample data set (targeting SMEs) sub-sample data set (targeting medium-sized firms). A paper that investigated the effects of inter-firm linkages on SME's innovation performance was published in Technological Forecasting and Social Change [1].

Descriptive statistics of the date file can be found in Table 1, Table 2, Table 3, Table 4.

**Table 1:** Distribution by the firm ownership.

	Frequency	Percent	Valid Percent	Cumulative Percent
Non-private	87	20.7	20.7	20.7
Private	333	79.3	79.3	100.0
Total	420	100.0	100.0

**Table 2:** Distribution by the firm export orientation.

	Frequency	Percent	Valid Percent	Cumulative Percent
No export	156	37.1	37.1	37.1
Do export	264	62.9	62.9	100.0
Total	420	100.0	100.0

**Table 3:** Distribution by the industries.

	Frequency	Percent	Valid Percent	Cumulative Percent
Bio Instruments and Equipment Industry	25	6.0	6.0	6.0
Electronics Equipment Industry	50	11.9	11.9	17.9
Hardware & Software Industry	27	6.4	6.4	24.3
Light Industry	155	36.9	36.9	61.2
Machinery Industry	123	29.3	29.3	90.5
Textiles Industry	40	9.5	9.5	100.0
Total	420	100.0	100.0

**Table 4:** Distribution by the firm size.

	Frequency	Percent	Valid Percent	Cumulative Percent
Medium- sized	319	76.0	76.0	76.0
Small-sized	101	24.0	24.0	100.0
Total	420	100.0	100.0	

### 1.1 Distribution by the firm ownership

Table 1 shows that most of the SMEs in the dataset were private firms (79.3%).

### 1.2 Distribution by the firm export orientation

Table 2 shows that most of the SMEs in the dataset did export on international market (62.9%).

### 1.3 Distribution by the industrial background of firms

Table 3 shows that six types of manufacturing industries were covered by the sampling firms, involving Bio Instruments and Equipment Industry (6.0%), Electronics Equipment Industry (11.9%), Hardware & Software Industry (6.4%), Light Industry (36.9%), Machinery Industry (29.3%), and Textiles Industry (9.5%).

### 1.4 Distribution by the firm size

Table 4 shows the distribution of the firm size, indicating that most of the firms were medium-sized firms (76.0%), while small part of them were small-sized firms (24.0%).

## 2 Experimental design, materials and methods

### 2.1 Ethical statements

The research was approved by the Research Ethics Committee of Tsinghua University and was carried out in accordance with the code of ethics governing questionnaire surveys.

### 2.2 Data collection

The data was collected via a questionnaire survey in Zhejiang Province, China.

The date was filtered based on the requirements of the statistical analysis method (i.e. structural equation modelling). We only kept the variables that are relevant to the analysis in the data file and removed other irrelevant variables. The filtering was carried out manually by using SPSS.

Specific variables in the data file were derived in the following ways:

Firm ownership was calculated by the item (Private), firm scale was calculated by the item (Person) with the item (lnperson) be transferred by natural logarithm algorithm, firms’ export orientation was measured by the item (export).

IP was calculated by set (p1-p4), representing the innovation performance of firms.

LPO presented in the full model was calculated by set (a1-a4), representing the linkages with competitors, suppliers, lead users and customers, and complementors; LSI was calculated by set (a5-a8), representing the linkages with universities, research institutes, governance agencies, and finance and law service agencies.

AC in one model was calculated by the standardized value of R&D expenditures (represented by ZRD), and in the other mode directly calculated by the item (IT), indicating the firm engaging information technology activities or not.

The item (industry) represented the industry background of samples.

Upon the measurements above, general statistical analysis approaches were used via software SPSS, which involve descriptive analysis, factor analysis, reliability and validity tests, and regression analysis. In addition, Structural Equation Model was adopted for data analysis via statistical software AMOs.

## References

[1] Mei L., Zhang T., Chen J. (2019). Exploring the effects of inter-firm linkages on SMEs' open innovation from an ecosystem perspective: an empirical study of Chinese manufacturing SMEs. Technol. Forecast. Soc. Chang..

