# Supplementary material for: Survey data on inter-firm linkages and innovation activities of Chinese manufacturing SMEs
Source: Data Brief. 2019 Oct 16;27:104671. doi: 10.1016/j.dib.2019.104671 (PMC6838382; doi:10.1016/j.dib.2019.104671)
Supplement: Multimedia component 1 [file mmc1.docx]

**The Survey Questionnaire**

This survey collects information on your enterprise’s innovations during the three years 2011 to 2013 inclusive. The next sections cover the general information, innovation performance, inter-firm linkages of your enterprise.

Please complete all questions as many as possible.

We will keep your information as secret and the result of data analysis is only for research.

Please tell us your general information, or enclose with your business name card.

**Your enterprise’s name：**

**Job Title:**

**Gender: 1. Male ( ) 2. Female ( )**

**Age: 1. 30 or below ( ) 2. 31 ~ 40 ( ) 3. 41 ~ 50 ( ) 4. 51 or above ( )**

**Working period：( ) years**

**How you know your company based on your working period and experience:**

| Not at all familiar | Low familiar | Slightly familiar | Neutral | Moderately familiar | Very familiar | extremely familiar |
| --- | --- | --- | --- | --- | --- | --- |
| 1 | 2 | 3 | 4 | 5 | 6 | 7 |

Person we should contact if there are any queries regarding the form:

Name: _____________________________________

Job title: _____________________________________

Phone: _____________________________________

E-mail: _____________________________________

# 1. General information about the enterprise

### The property of your enterprise

A、private ( ) B、non-private ( )

### The Year of your enterprise’s establishment: ______________

### Total number of employees in your enterprise: ______________

### Number of employees with graduate education or a scientific-technical graduate education: ______________

### Does the enterprise export products abroad in recent three years?

A、Yes ( ) B、No ( )

### Does your enterprise have been involved in information activities as follows? :

A、adopting advanced production system (CIMS, ERP, and so on) (1. Yes 2. No)

B、promoting IT-based communication and knowledge management system (1. Yes 2. No)

C、firm-level information management platform (1. Yes 2. No)

D、others ______________

### According to the main business, what industry does your enterprise belongs to? (you could list the main business of your enterprise instead if unclear about the industry setting): ______________

### In which city(town) is the head office of your enterprise located? : _____________

## 2. Innovation Performance

Rate the extent to which your enterprise was successful relative to your major competitors in terms of the following items

| In recent three years, our company | extremely disagree | Very disagree | Moderately disagree | Neutral | Moderately agree | Very agree | extremely agree |
| --- | --- | --- | --- | --- | --- | --- | --- |
| Frequently introducing new products | 1 | 2 | 3 | 4 | 5 | 6 | 7 |
| Being first in new product introductions in the market | 1 | 2 | 3 | 4 | 5 | 6 | 7 |
| Quickly launching new products into the market | 1 | 2 | 3 | 4 | 5 | 6 | 7 |
| Developing new products with superior quality | 1 | 2 | 3 | 4 | 5 | 6 | 7 |

## 3. Inter-firm Linkages

Rate the extent to which your firm has close linkage with in recent three years

| In recent three years, our company | Not at all important | Low important | Slightly important | Neutral | Moderately important | Very important | extremely important |
| --- | --- | --- | --- | --- | --- | --- | --- |
| Competitors | 1 | 2 | 3 | 4 | 5 | 6 | 7 |
| Suppliers for components | 1 | 2 | 3 | 4 | 5 | 6 | 7 |
| Lead users and customers | 1 | 2 | 3 | 4 | 5 | 6 | 7 |
| Complementor | 1 | 2 | 3 | 4 | 5 | 6 | 7 |
| Universities | 1 | 2 | 3 | 4 | 5 | 6 | 7 |
| Research institutes | 1 | 2 | 3 | 4 | 5 | 6 | 7 |
| Government agencies | 1 | 2 | 3 | 4 | 5 | 6 | 7 |
| Finance and law service agencies | 1 | 2 | 3 | 4 | 5 | 6 | 7 |
